# The maxillary canal of the titanosuchid *Jonkeria* (Synapsida, Dinocephalia)

**DOI:** 10.1007/s00114-023-01853-w

**Published:** 2023-06-05

**Authors:** Julien Benoit, Luke A. Norton, Sifelani Jirah

**Affiliations:** Evolutionary Studies Institute, University of the Witwatersrand, Johannesburg, South Africa

**Keywords:** Dinocephalia, Trigeminal, Maxillary canal, Sections, Behaviour, Phylogeny

## Abstract

The maxillary canal of the titanosuchid dinocephalian *Jonkeria* is described based on digitised serial sections. We highlight that its morphology is more like that of the tapinocephalid *Moschognathus* than that of *Anteosaurus*. This is unexpected given the similarities between the dentition of *Jonkeria* and *Anteosaurus* (i.e., presence of a canine) and the fact that the branching pattern of the maxillary canal in synapsids usually co-varies with dentition. Hypotheses to account for similarities between *Jonkeria* and *Moschognathus* (common ancestry, function in social signalling or underwater sensing) are discussed. It is likely that the maxillary canal carries a strong phylogenetic signal, here supporting the clade Tapinocephalia.

**Supplementary Information:**

The online version contains supplementary material available at 10.1007/s00114-023-01853-w.

## Introduction

In basal synapsids, the trigeminal nerve and accompanying vessels were carried through the maxillary bone by a canal called the maxillary canal (Benoit et al. 2016a, b, 2017a, b, 2018, 2020, 2021a, b; Miyamae and Bhullar 2017). This canal is at least partly homologous to the superior alveolar canal of reptiles and the infraorbital foramen of mammals (Benoit et al. 2021a). Descriptions of the maxillary canal in basal synapsids used to be scarce in the literature but their number increased tremendously in recent years. It is now documented in some pelycosaurs (Benoit et al. 2021a), the basal-most therapsid *Raranimus* (Duhamel et al. 2021), two dinocephalians (Benoit et al. 2021b), some gorgonopsians and biarmosuchians (Benoit et al. 2016a), several dicynodonts (Laaß and Kaestner 2017; Benoit et al. 2018; Araujo et al. 2022), therocephalians (Benoit et al. 2016b, 2017b; Pusch et al. 2020) and non-mammalian cynodonts (Benoit et al. 2016a, 2020; Pusch et al. 2019; Wallace et al. 2019; Franco et al. 2021). These works have helped refine knowledge on non-mammalian synapsid phylogeny and palaeobiology and explored new hypotheses about their sensory evolution, social behaviour, and the development of a venomous bite in therocephalians, a keratinous beak in dicynodonts, and sensory vibrissae in the lineage leading to mammals. Here, we contribute to this growing body of work by describing for the first time the maxillary canal of the titanosuchid dinocephalian *Jonkeria truculenta*.

## Material and methods

Data on the maxillary canal of large animals such as titanosuchid dinocephalians are notoriously difficult to obtain as their skull does not fit into regular micro-CT machines. As such, here the data were acquired thanks to the digitisation of the physical sections of specimen SAM-PK-11575. Boonstra (1962) identified SAM-PK-11575 as *Jonkeria ingens*, which has recently suggested to be a junior synonym of *Jonkeria truculenta* (Jirah 2022).

The sections of SAM-PK-11575 were photographed using a DSLR camera mounted on a tripod. The resulting images were processed using GIMP and manually aligned using SPIERSalign (Sutton et al. 2012). Slice intervals were estimated to be ~5 mm for the three-dimensional (3-D) reconstruction. A more detailed description of the workflow is described in the supplementary information.

The maxillary canal and teeth were manually segmented using Avizo 9 (Thermo Fisher Scientific, Hillsborough, OR, USA) as if the data were obtained through CT-scanning. The resulting 3-D model is compared to the maxillary canals of *Anteosaurus magnificus* (BP/1/7074) and *Moschognathus whaitsi* (AM4950) (Benoit et al. 2021b).

Institutional abbreviations are ad follows: AM: Albany Museum (Grahamstown, South Africa), BP: Evolutionary Studies Institute (Johannesburg, South Africa); SAM: Iziko: South African Museum (Cape Town, South Africa).

### Description and comparison

**Fig. 1:**
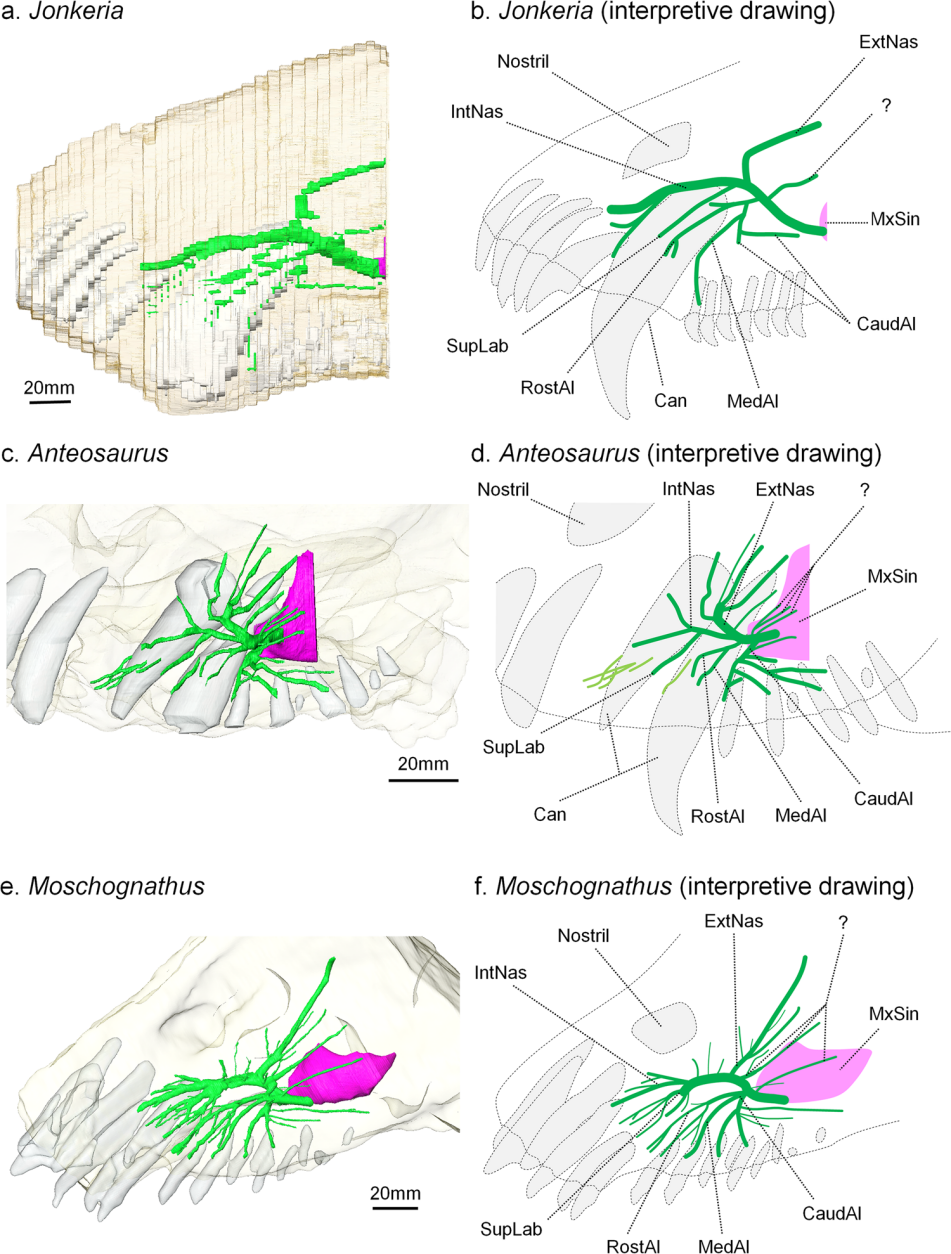
The maxillary canal system of dinocephalians in lateral view. **a** *Jonkeria truculenta* (SAM-PK-11575); **b** Interpretive drawing of **a**; **c**, *Anteosaurus magnificus* (BP/1/7074); **d**, interpretive drawing of **c**; **e**, *Moschognathus whaitsi* (AM4950); **f**, interpretive drawing of **e**. Green, maxillary canal; purple, maxillary sinus; white, teeth. Abbreviations: Can, canine (caniniform tooth); CaudAl, caudal alveolar canal; ExtNas, external nasal canal; IntNas, internal nasal canal; MedAl, medial alveolar canal; MxSin, maxillary sinus; RosAl, rostral alveolar canal; SupLab, superior labial canal

Though the raw reconstruction of the maxillary canal is jagged in lateral view (Fig. 1a), all the branches are distinctly identifiable (Fig. 1b). As the maxillary canal is a mostly two-dimensional structure (Benoit et al. 2016a), it is here described in lateral view only.

Overall, the maxillary canal of *Jonkeria* is a lot less branched than in *Moschognathus* and *Anteosaurus* (Fig. 1a, b). In *Moschognathus*, the branching is more intense rostrally, whereas in *Anteosaurus*, the branching is concentrated caudally (Fig. 1c, d). Like in *Moschognathus*, the maxillary canal of *Jonkeria* is divided between a very thick, rostrocaudally oriented main trunk, and thinner peripheral branches. In contrast, all branches are of similar thickness in *Anteosaurus* (Fig. 1c). The main trunk bends dorsally at the level of the canine socket in *Jonkeria* and *Anteosaurus*. Noticeably, this bending is also present in *Moschognathus* despite the absence of a caniniform tooth (Fig. 1).

In *Jonkeria*, the internal nasal canal is oriented mostly rostroventrally and branches into three smaller canals rostral to the canine (Fig. 1a). Unlike in *Anteosaurus*, there is no branch oriented dorsally on the internal nasal canal. Just ventrally, the superior labial canal runs parallel to the internal nasal canal in *Jonkeria*. Unlike in *Moschognathus*, it is not branched. This condition is similar to that in *Anteosaurus* and most therapsids (Benoit et al. 2016a).

In *Jonkeria*, the external nasal canal branches off more caudally, at the level of the distal margin of the canine socket (Fig. 1). It is oriented dorsally and caudally and consists of a long, thick and unbranched canal. This is more similar to the condition in *Moschognathus*, in which the canal is also long, thick and branches into few minor canals, whereas in *Anteosaurus* it branches into four large canals (Fig. 1). The condition in *Anteosaurus* is the most common among synapsids (Benoit et al. 2016a, 2018). The long external nasal canal strongly bends caudally in *Jonkeria*, forming an almost 90° angle (Fig. 1a, b). This differs from the straight canal in *Moschognathus*.

Immediately caudal to the external nasal canal, a small dorsally oriented canal branches off the main trunk (labelled “?” in Fig. 1), and this branch is unidentified. *Anteosaurus* and *Moschognathus* possess respectively three and four branches in a similar position. An unidentified canal in a similar position—but perhaps not homologous—is also present in the therocephalians *Bauria* and *Olivierosuchus* (Benoit et al. 2016b, 2017b) and the anomodont *Patranomodon* (Benoit et al. 2018).

Ventrally, the alveolar canals in *Jonkeria* are not strongly branched (Fig. 1a, c). The rostral alveolar canals are directed towards the base of the canine in *Jonkeria*, as is usual for therapsids (Benoit et al. 2020). This canal is divided into two branches only, as in *Anteosaurus*, although one is markedly shorter than the other. In *Moschognathus*, the rostral alveolar canal is strongly branched. The median alveolar canals are directed towards the first postcanine tooth in *Jonkeria*, as in *Moschognathus*. In *Anteosaurus*, this branch is directed towards the base of the canine (Fig. 1). In *Jonkeria*, the caudal alveolar canal is markedly simpler than in *Moschognathus* and *Anteosaurus*, as it divides into two branches only. One of these branches is oriented vertically towards the postcanine teeth, and the other is oriented horizontally, parallel to the tooth row. A similar horizontal caudal alveolar branch is also present in *Anteosaurus* and *Moschognathus* (Fig. 1), as well as in *Raranimus*, *Olivierosuchus* and the gorgonopsians, biarmosuchians and pelycosaurs for which the maxillary canal has been described (Benoit et al. 2016a, 2018; Duhamel et al. 2021). This is likely a plesiomorphic condition for synapsids (Duhamel et al. 2021). Unlike in pelycosaurs and *Raranimus*, this horizontally running branch does not give off lateral branches at a regular interval (Duhamel et al. 2021). Only the rostralmost end of the maxillary sinus is preserved, which prevents description.

## Discussion

The maxillary canal in *Jonkeria* differs from those in the other two known dinocephalian as it uniquely combines a distinctly thickened main trunk and unbranched internal and external nasal canals, as in *Moschognathus*, with an unbranched superior labial canal as in *Anteosaurus* (Fig. 1). The alveolar canals are also relatively unbranched compared to described maxillary canals in gorgonopsians, biarmosuchians, *Raranimus* and pelycosaurs (Benoit et al. 2016a, 2018; Duhamel et al. 2021).

*Jonkeria* and *Moschognathus* belong to the Titanosuchidae and Tapinocephalidae, respectively, which together form the clade Tapinocephalia (Hopson and Barghusen 1986). Given this, it is noteworthy that the structure of the maxillary canal in *Jonkeria* shares more similarities with that of *Moschognathus* rather than with that of *Anteosaurus*. The thickening of the main trunk of the maxillary canal and unbranched condition of the external nasal canal are unique to *Jonkeria* and *Moschognathus* among synapsids (Benoit et al. 2016a, 2018; Duhamel et al. 2021). In contrast, the unbranched superior labial canal that *Jonkeria* and *Anteosaurus* share is present in pelycosaurs and *Raranimus* and is a common, likely plesiomorphic condition among other therapsids (Benoit et al. 2016a; Duhamel et al. 2021).

As the maxillary canal gives passage to the sensory fibres of the maxillary branch of the trigeminal nerve, its variations in tetrapods are usually interpreted as reflecting adaptations of facial sensitivity to various stimuli, most often tactile ones (e.g., Benoit et al. 2016a; Bouabdellah et al. 2022; Lessner et al. 2023). In this respect, titanosuchids and tapinocephalids shared similar ecology and physiology that may account for the similarities in their maxillary canal morphology. This includes a semi-aquatic lifestyle (Bhat et al. 2022), which can shape the morphology of the rostral vascular system to help sensing water pressure variations (Lessner et al. 2023). Facial sensitivity is also used for social interactions (Grant and Goss 2022). Tapinocephalid skeletons have often been found in groups comprising five to twelve individuals (Gregory 1926; Boonstra 1955; Rubidge et al. 2019; Almond 2022), and Boonstra (1962) reported that SAM-PK-11575 was found alongside two other *Jonkeria* skulls. As such, it is likely that titanosuchids and tapinocephalids lived in small groups, at least for part of the year, perhaps during the reproductive season.

As a final observation, it is remarkable that despite the absence of an enlarged caniniform tooth, *Moschognathus* still displays a slight dorsal bending of the main trunk of the maxillary canal above the canine socket, as in *Anteosaurus* and *Jonkeria* (Fig. 1). This illustrates how phylogenetically conservative the morphology of the maxillary canal can be and suggests that the maxillary canal could be used as a valuable source of phylogenetic characters (see, e.g., Duhamel et al. 2021; Benoit et al. 2021a, 2022). This previously out-of-reach structure therefore holds promises for helping to resolve some notoriously long-standing problems in synapsid phylogeny.

## Supplementary information

ESM 1 Additional file 1: SI1 Method used to digitised SAM-PK-11575 serial sections. (PDF 1.12 MB)
