# Supplementary material for: The maxillary canal of the titanosuchid Jonkeria (Synapsida, Dinocephalia)
Source: Naturwissenschaften. 2023 Jun 5;110(4):27. doi: 10.1007/s00114-023-01853-w (PMC10241669; doi:10.1007/s00114-023-01853-w)
Supplement: Supplementary file 1 — Additional file 1: SI1 Method used to digitised SAM-PK-11575 serial sections. (PDF 1.12 MB) [file 114_2023_1853_MOESM1_ESM.pdf]

The maxillary canal of the titanosuchid *Jonkeria* (Synapsida, Dinocephalia)

Julien Benoit[1*], Luke A. Norton[1] & Sifelani Jirah[1]

[1]Evolutionary Studies Institute, University of the Witwatersrand, Johannesburg, South Africa
[*]Corresponding author: julien.benoit@wits.ac.za

Supplementary Information

Institutional Abbreviations

AM Albany Museum, Makhanda (Grahamstown), South Africa
BP Evolutionary Studies Institute, University of the Witwatersrand, Johannesburg, South Africa
SAM Iziko: South African Museum, Cape Town, South Africa

Material

Specimen SAM-PK-11575 was recovered by L.D. Boonstra from a sandstone layer on Klein-Koedoeskop 310, Beaufort West District (Boonstra 1962), which is situated within the *Tapinocephalus* Assemblage Zone of the Main Karoo Basin (Day and Rubidge 2020). According to Boonstra (1962) the specimen was serially cross-sectioned for the study of internal structures (see Boonstra 1968).

What remains of SAM-PK-11575 is preserved as a series of 31 sequential coronal sections and a single sagittal section through the snout. Each coronal section exposes two faces (except the first), resulting in a series total of 61 slices. The average thickness of each physical section is approximately 0.6 mm. The slices were digitised and reconstructed following the procedure described below.

Methods

Photography

Sections were photographed using a Canon EOS 450D fitted with a Canon EF-S 15–85 mm f/3.5–5.6 IS USM lens mounted to a tripod. Image stabilisation (IS) was disabled, and a hot-shoe mounted spirit level was used to ensure that the image sensor was level. A remote shutter release (Canon RS-60E3) was used to actuate the shutter without the need to handle the camera set up between photographs. The working distance between image sensor and specimen was kept as consistent as possible; however slight variation may have occurred due to differences in slice thickness. For all photographs the ISO and aperture were set at 100 and *f*/16, respectively. Shutter speed varied from 0.5–1 second.

Image Processing

The raw JPG images (4272 × 2848 pixels) were imported into GIMP (https://gimp.org) for processing (e.g., Fig. S1a–c). Due to the nature of the sections, each alternate photograph needed to be flipped/mirrored for the anatomical structures to be in the same orientation (e.g., Fig. S1b). Some images needed to be rotated 90° as

they were photographed in a different orientation. The resulting image stack was cropped to reduce the file sizes. A black background layer was inserted at the bottom of the image stack so that after rotating and cropping, the resulting images would have a consistent image size of 3035 × 3181 pixels. In addition, a black mask was applied to the area surrounding the specimen in order to removed excess ‘noise’ (e.g., accumulated dust and cloth background). Note that noise removal is optional, but can improve the results of automated ‘thresholding’ in the later steps (e.g., segmentation). No other processing (e.g., colour correction, image sharpening, etc.) was applied to the images. The processed image stack was exported as 61 individual PNG files (e.g., Figs S1d–f).

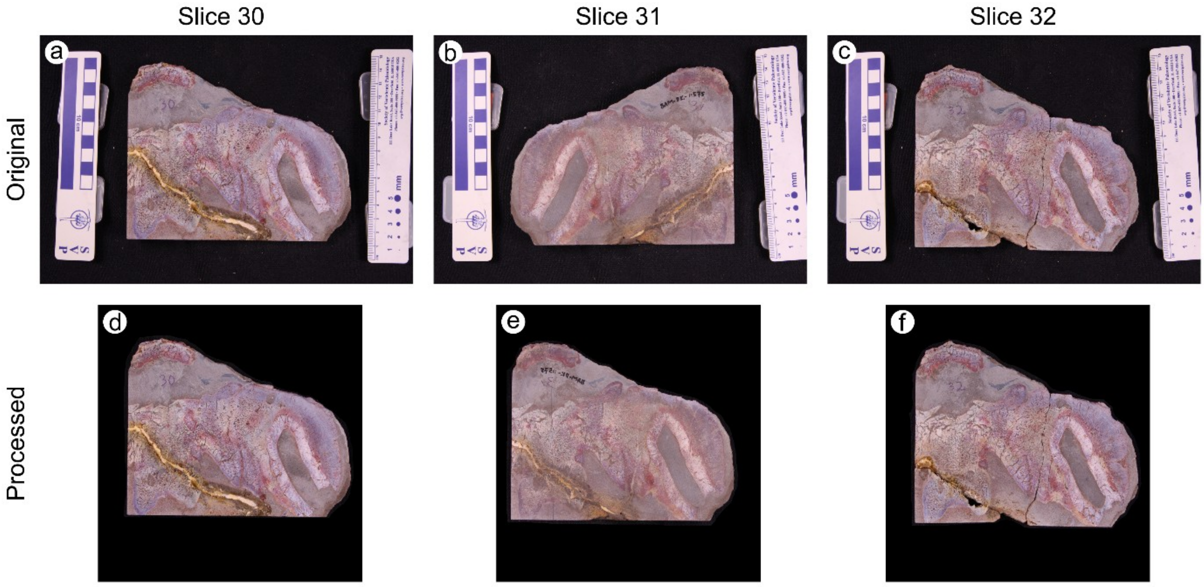

Fig. S1 Image processing workflow showing three sequential slices of *Jonkeria truculenta* (SAM-PK-11575). a–c, original images (4272 × 2848 pixels); d–f, processed images showing re-orientation of slice 31 (e), cropped area (3035 × 3181 pixels), and removal of excess detail surrounding specimen. Note Slice 30 and Slice 31 represent opposing faces of the same physical section

Image Alignment

The PNG stack was loaded into SPIERSalign V 3.1.0 (Sutton et al. 2012; https://spiers-software.org). Fiduciary markers were set on the straight edges of the middle image (Slice 31; Fig. S2). Alignment was performed manually, using the ‘Rotate’ and ‘Shift’ commands from the Transform dropdown menu. A ‘first pass’ through the image stack ensured that the region of interest for all 61 images would remain within the 3035 × 3181 pixel limit (orange vertical markers in Fig. S2). A ‘second pass’ was then undertaken to refine the rotation of the images, aligning them first to the blue horizontal marker, and then to the orange vertical marker on the right (Fig. S2). The stack of aligned images was cropped in SPIERSalign, and the resulting images were exported as TIF files.

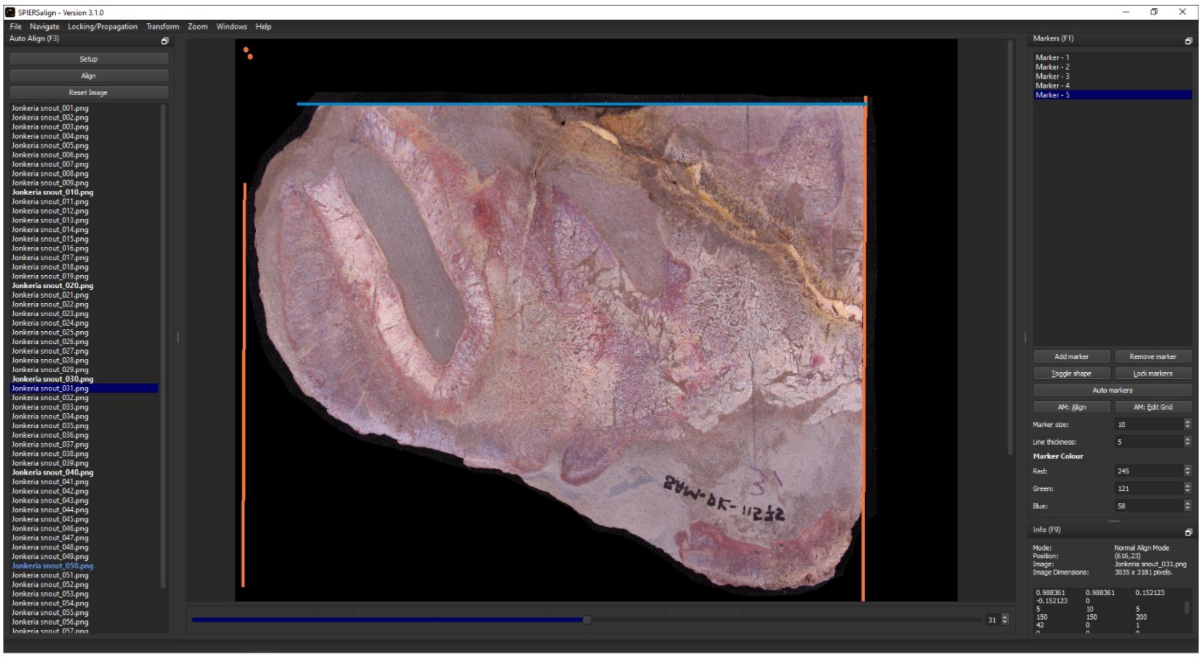

Fig. S2 User interface of SPIERSalign V 3.1.0 (Sutton et al. 2012). Note: SPIERSalign requires a minimum of five fiduciary markers, hence the two 'unused' circular markers visible in the top left corner

Reconstruction

The TIF stack was loaded into 3D Slicer (Kikinis et al. 2014; https://slicer.org) using the 'ImageStacks' function within the SlicerMorph extension (Rolfe et al. 2021; https://slicermorph.github.io). We are unsure of the thickness of the disc used to create the sections. A modest estimate of 4 mm was used, thus the volume was reconstructed with a slice interval of 6 mm. The reconstructed volume was exported as an NRRD file.

Segmentation

The maxillary canal and teeth were manually segmented using the commercially available software Avizo 9 (Thermo Fisher Scientific, Hillsborough, OR, USA). Similar results could be obtained from using the SPIERSedit module, or other 3-D visualisation software (see Buser et al. 2020). Screenshots of the final segmented data were exported as TIF images (Fig. 1).

Comment on 'Resolution' of Reconstructions

The focus of this study was to describe the gross morphology of the maxillary canals in a serially sectioned specimen of *Jonkeria truculenta*, with particular interest in the positions of the various branches of these canals relative to other identifiable anatomical structures (e.g., canine alveolus, etc.). Although the reconstructed slice interval of 6 mm is of a relatively low resolution by modern standards (cf. 0.102 mm voxel size achieved from X-ray microtomography (Benoit et al. 2021)). However, the large size of these animals (skull length of *Jonkeria* ~520–680 mm (Jirah 2022)), means that this slice interval is adequate for recording sufficient morphology to make the description of the maxillary canals feasible. As such, the 'jagged' appearance of the raw 3-D reconstruction has a negligible effect on the descriptions in the main text.

References

Benoit J, Kruger A, Jirah S, Fernandez V, Rubidge BS (2021) Palaeoneurology and palaeobiology of the dinocephalian therapsid *Anteosaurus magnificus*. Acta Palaeontol Pol 66:29–39. https://doi.org/10.4202/app.00800.2020

Boonstra LD (1962) The dentition of the titanosuchian dinocephalians. Ann S Afr Mus 46:57–112

Boonstra LD (1968) The braincase, basicranial axis and median septum in the Dinocephalia. Ann S Afr Mus 50:195–273

Buser TJ, Boyd OF, Cortés Á, et al. (2020) The natural historian's guide to the CT galaxy: step-by-step instructions for preparing and analyzing computed tomographic (CT) data using cross-platform, open access software. Integr Org Biol 2:obaa009. https://doi.org/10.1093/iob/obaa009

Day MO, Rubidge BS (2020) Biostratigraphy of the *Tapinocephalus* Assemblage Zone (Beaufort Group, Karoo Supergroup), South Africa. S Afr J Geol 123:149–164. https://doi.org/10.25131/sajg.123.0012

Jirah S (2022) Middle Permian diversity of large herbivores: taxonomic revision of the Titanosuchidae (Therapsida, Dinocephalia) of the Karoo Basin, South Africa. Ph.D. thesis, University of the Witwatersrand

Kikinis R, Pieper SD, Vosburgh KG (2014) 3D Slicer: A Platform for Subject-Specific Image Analysis, Visualization, and Clinical Support. In: Jolesz F (ed) Intraoperative Imaging and Image-Guided Therapy. Springer, New York, pp 277–289

Rolfe S, Pieper S, Porto A, et al. (2021) SlicerMorph: An open and extensible platform to retrieve, visualize and analyze 3D morphology. Methods Ecol Evol 12:1816–1825. https://doi.org/10.1111/2041-210X.13669

Sutton MD, Garwood RJ, Siveter DJ, Siveter DJ (2012) SPIERS and VAXML; a software toolkit for tomographic visualisation and a format for virtual specimen interchange. Palaeontol Electron 15.2.5T: https://doi.org/10.26879/289
